# Causal associations between urinary sodium with body mass, shape and composition: a Mendelian randomization study

**DOI:** 10.1038/s41598-020-74657-x

**Published:** 2020-10-15

**Authors:** Qi Feng, Shuai Yuan, Qian Yang, Yingchang Lu, Ruth J. F. Loos, Gloria H. Y. LI, Yue Fei, Man Fung Tsoi, Ching Lung Cheung, Bernard M. Y. Cheung

**Affiliations:** 1 Department of Medicine, LKS Faculty of Medicine, Queen Mary Hospital, The University of Hong Kong, Pokfulam, Hong Kong China; 2 Unit of Cardiovascular and Nutritional Epidemiology, Institute of Environmental Medicine, Karolinska Institute, Stockholm, Sweden; 3 Medical Research Council Integrative Epidemiology Unit, University of Bristol, Bristol, UK; 4 Population Health Sciences, University of Bristol, Bristol, UK; 5 Vanderbilt Genetics Institute, Vanderbilt University Medical Center, Nashville, TN USA; 6 The Charles Bronfman Institute for Personalized Medicine at Mount Sinai, Icahn School of Medicine at Mount Sinai, New York, NY USA; 7 Department of Pharmacology and Pharmacy, LKS Faculty of Medicine, The University of Hong Kong, Pokfulam, Hong Kong China; 8 Centre for Genomic Sciences, LKS Faculty of Medicine, The University of Hong Kong, Pokfulam, Hong Kong China; 9 State Key Laboratory of Pharmaceutical Biotechnology, The University of Hong Kong, Pokfulam, Hong Kong China; 10 Institute of Cardiovascular Science and Medicine, The University of Hong Kong, Pokfulam, Hong Kong China

**Keywords:** Medical research, Risk factors

## Abstract

Observational studies have found associations between urinary sodium (UNa) with obesity, body shape and composition; but the findings may be biased by residual confounding. The objective of this two-sample Mendelian randomization (MR) study was to analyze their causal associations in both sex-combined and sex-specific models. Genome-wide association studies of UNa, body mass index (BMI), BMI-adjusted waist-to-hip ratio (WHR), body fat (BF) percentage and estimated glomerular filtration rate (eGFR) were identified. We initially extracted fifty SNPs associated with UNa at significance level of 5 × 10^–8^, but further removed those SNPs with potential horizontal pleiotropy. Univariable and multivariable MR with adjustment for eGFR were performed. Inverse-variance weighted MR was performed as the primary analysis, with MR-Egger methods as sensitivity analysis. The potential bidirectional association between BMI and UNa was investigated. All exposure and outcomes were continuous, and the effect measure was regression coefficients (beta) and their 95% confidence intervals (95% CI). The total sample size was up to 322 154. UNa was causally associated with increased BMI in both men [eGFR-adjusted beta 0.443 (0.163–0.724)] and women [0.594 (0.333–0.855)]. UNa caused BF percentage increase in men [0.622 (0.268–0.976)] and women [0.334 (0.007–0.662)]. UNa significantly elevated BMI-adjusted WHR in men [0.321 (0.094–0.548)], but not in women [0.170 (− 0.052 to 0.391)]. Additionally, we found that BMI causally increased UNa [0.043 (0.023–0.063)]. UNa increased BMI and BF percentage. Salt intake affects male body shape by increasing BMI-adjusted WHR, but showed no effects on female body shape. The bidirectional association between BMI and UNa suggested that salt reduction measures and weight reduction measures should be implemented simultaneously to break the vicious cycle and gain more health benefits.

## Introduction

Obesity has been a global public health concern with increasingly substantial disease burdens^1^. Obesity is believed to result from a complex relationship among psychosocial, biological and behavioral factors, such as genetic susceptibility, excessive calorie intake and physical inactivity^2^. Other factors also contribute independently to development of obesity^2,3^. Obesity, commonly measured with body mass index (BMI, body weight in kilogram/squared height in meter), is a well-recognized risk factor for a range of health conditions and diseases, including cardiovascular diseases^4^, diabetes^5^ and cancers^6^. BMI is easy to measure and calculate; nevertheless, body shape and body composition are stronger predictors for health outcomes than BMI^7,8^.

Dietary salt intake and urinary sodium (UNa) are known to be associated with a range of health outcomes, such as hypertension, cardiovascular diseases and death^9–11^. Urinary sodium is a surrogate measure for salt intake^12^. Observational studies^13–17^ have suggested their effects on obesity. For example, a systematic review of observational studies^13^ found that both dietary salt intake and UNa were positively associated with BMI. A recent cross-sectional study^14^ showed that this positive association was directionally concordant across Asian and Western populations, with varying effect sizes. Furthermore, UNa was also demonstrated to be correlated with body shape and body composition measures, such as waist circumference (WC)^15,16^, waist-to-hip ratio (WHR, waist circumference/hip circumference)^16^, body fat (BF) mass and lean mass^17^.

However, so far, the majority of the evidence has been generated from observational studies, mainly cross-sectional studies, in which residual confounding and reverse causation are likely. Evidence from randomized controlled trials has been limited. Although there have been many randomized trials investigating the effects of salt reduction on health outcomes^18,19^, very few of them reported obesity-related outcomes. Furthermore, they were flawed with small sample size, short follow-up and restricted generalizability^20^. Mendelian randomization (MR), by using single nucleotide polymorphisms (SNPs) as instrumental variables to investigate the causal link between two phenotypes, is helpful in causal inference, because it removes reverse association and residual confounding^21^. The objective of this study is to examine the causal association between UNa with body mass, shape and composition using two-sample MR approach.

## Methods

We used summary-level statistics from relevant GWA studies, where ethical approval and patient consent had been obtained. The exposure of interest was UNa. The outcomes of interest included BMI, body shape and body composition. For body shape, we used BMI-adjusted WHR as the primary outcome, while WC, hip circumference (HC), WHR, BMI-adjusted WC, BMI-adjusted HC were secondary outcomes. For body composition, we used BF percentage as the primary outcome, while whole-body lean mass (WLM) and appendicular lean mass (ALM) as secondary outcomes. Since sex has been suggested as a potential effect modifier in obesity-related outcomes^22,23^, we further investigated sex-specific effects in men and women separately.

### SNP selection

A GWA study including 446,237 European-ancestry individuals from UK Biobank^24^ identified 50 lead SNPs associated with UNa that reached significance level of 5 × 10^–8^ (Supplementary Table S1). Spot urine sample collection and storage in UK Biobank have been described elsewhere^11^. UNa concentration was measured by the ion-selective electrode method. The effect between genetic variants and log-transformed UNa concentration was adjusted for age, sex, array information and ancestral principal components. The SNPs are located in different gene regions and distributed independently (not in linkage disequilibrium), and explain 6.4% of the total variance of the trait^24^. If an SNP could not be matched in the GWA studies of an outcome, a proxy in linkage disequilibrium with the SNP (R^2^ > 0.80) would be identified; if no proper proxy was identified, the unmatched SNP would have to be removed from MR analysis. Relevant traits associated with the SNPs were also searched in the PhenoScanner v2 database at a significance level of 5 × 10^–8^ for potential pleiotropy.

### Outcome data

We extracted summary-level data of the outcomes from four GWA studies of BMI^25^, body shape^26^ and body composition^27,28^. The BMI GWA study^25^ included 322,154 European-ancestry individuals (152,882 men and 171,963 women) from 125 cohorts. The effect was adjusted for age, squared age, ancestral principal components and study-specific covariates. The body shape GWA study^26^ included totally 211,088 European-ancestry individuals (93,480 men and 116,742 women), and provided data for BMI-adjusted and unadjusted WC, HC and WHR, additionally adjusted for age, squared age and ancestry principal components. The BF percentage GWA study^28^ included 89,297 European-ancestry individuals (44,429 men and 45,525 women), and was adjusted for age, squared age and ancestral principal components. The GWA study for WLM and ALM^27^ included 38,292 and 28,330 European-ancestry individuals, respectively, and was adjusted for age, sex, squared age, height, body fat percentage and ancestral principal components. Body composition (BF, WLM and ALM) was measured either with bioimpedance analysis or dual-energy X-ray absorptiometry^26,27^. Sex-combined and sex-specific outcome data were available from the GWA studies of BMI, body shape and BF percentage. The GWA study for WLM and ALM provided sex-combined data only.

### Covariate

Renal function is associated with both UNa^29^ and obesity^30,31^, which is a potential confounder in the associations of interest. We identified a GWA study^32^ of estimated glomerular filtration rate (eGFR), which included 567,460 European-ancestry individuals. Linear regression of log-transformed eGFR was adjusted for age, sex and ancestry principal components.

### Statistical analysis

Previous studies^24^ have suggested that some of the UNa-associated SNPs are related to adiposity phenotypes, which could introduce horizontal pleiotropy and violate one of the underlying assumptions in MR analysis. To minimize potential horizontal pleiotropy, we further removed SNPs that: (1) were associated with the outcomes of interest at significance level of 5 × 10^–8^, or (2) were identified as outliers by the MR Pleiotropy RESidual Sum and Outlier (MR-PRESSO) outlier test^33^. Therefore, the included SNPs for each outcome actually differed but remained a subset of the 50 SNPs (or their proxies) identified by the UNa GWA study^24^. Supplementary file 1 shows the characteristics of the SNPs included in the MR analysis for each outcome. F statistic was used to measure the strength of instrumental variables, and an F statistic > 10 suggests strong instruments^34^.

All GWA summary-level data were harmonized with the method of Hartwig et al.^35^, so that the effect estimates of SNPs on all phenotypes were shown for the same alleles. Univariable MR analyses were performed with inverse-variance weighted method as primary analysis, while weighted median method and MR-Egger regression method as sensitivity analysis. Multivariable MR analyses adjusted for eGFR with inverse-variance weighted method were performed as primary analysis, while MR-Egger method as secondary analysis. The intercept test for MR-Egger regression was used to examine residual horizontal pleiotropy, with a P value < 0.05 suggesting pleiotropy.

To investigate sex-specific effects, we extracted male-specific and female-specific outcome data from the GWA studies^25,26,28^, and performed MR analyses separately. Sex-specific effects on WLM and ALM were not performed, because the data were not available. To examine the effect difference between men and women, we performed a heterogeneity test similar to that in meta-analysis, using Cochran’s Q test. A P value for Cochrane’s Q test < 0.10 indicates statistical significance.

To investigate potential bidirectional association between UNa and BMI, we also performed MR analysis with BMI as the exposure and UNa as the outcome. Ninety-seven BMI-associated SNPs were extracted from the BMI GWA study^25^, but 84 SNPs were finally included after removing those unmatched with UNa GWA data^24^ and/or identified as outliers by the MR-PRESSO outlier test. Similarly, univariable and multivariable MR analyses were performed.

A second way to deal with potential confounding effect of eGFR, alternative to multivariable MR, was to remove the SNPs associated with eGFR. We found that rs1260326 (located in *GCKR* gene) was associated with eGFR at a significance level of 5 × 10^–8^ in Phenoscanner v2 database; therefore, a sensitivity analysis by removing rs1260326 in univariable MR was performed as sensitivity analysis. Additional sensitivity analyses were performed by including outlier SNPs detected by the MR-PRESSO outlier tests. Since all outcomes were continuous, the effect measure was regression coefficient beta with its 95% confidence interval (CI), which should be interpreted as the change in units of standard deviation (SD) in the outcome when the log-UNa level increases 1 SD. Statistical significance was indicated by P value < 0.05. We did not apply Bonferroni correction, although we employed three primary outcomes, because their effects were measured from three independent samples of individuals, instead of one single sample. All statistical analysis was conducted with “*MendelianRandomization*” (version 0.4.1. https://CRAN.R-project.org/package=MendelianRandomization) and “*MRPRESSO*” (version 1.0. https://rdrr.io/github/rondolab/MR-PRESSO/) packages in R environment (R Core Team, 2003. https://www.R-project.org ).

We planned to perform power calculations based on the method of Brion et al.^36^, which required the beta estimates from our study and another beta estimates from ordinary least squares estimation (i.e., observational analysis). However, the UNa GWA study analyzed UNa concentration after log transformation (in log-mmol/L), while previous observational studies analyzed UNa concentration in a natural unit (mmol/L) or converted it to 24-h UNa (mmol/day or g/day) or daily salt intake (g/day), thus making it impossible to make direct comparison of the two beta estimates in the absence of individual participant data.

## Results

The proportions of exposure variance explained by the instrument variables ranged from 4.036 to 5.565% (mean 4.780%), and the according F statistics ranged from 536.146 to 613.340 (mean 587.078), suggesting strong instrumental variables (Table 1). Supplementary Information S1 showed detailed information on the SNPs included in the MR analysis for each outcome.

**Table 1:** Results of univariable Mendelian randomization analyses of sex-combined and sex-specific associations between urinary sodium secretion with body mass, shape and composition outcomes.

Outcome	SNPs	N	R^2^ (%)	F statistic	Inverse-variance weighed method		Weighted median methods		MR-Egger method		
					Beta (95% CI)	P for beta	Beta (95% CI)	P for beta	Beta (95% CI)	P for beta	P for Egger intercept
**Sex-combined**											
BMI	34	322,154	4.227	579.157	0.392 (0.149, 0.634)	0.002	0.542 (0.287, 0.797)	< 0.001	− 0.193 (− 1.387, 1.001)	0.751	0.327
HC	35	211,088	4.036	536.146	0.247 (− 0.014, 0.509)	0.064	0.215 (− 0.080, 0.510)	0.153	− 0.286 (− 1.703, 1.131)	0.693	0.453
WC	37	211,088	4.546	574.298	0.508 (0.228, 0.789)	< 0.001	0.526 (0.231, 0.822)	< 0.001	− 0.041 (− 1.452, 1.370)	0.954	0.436
WHR	38	211,088	4.703	579.443	0.449 (0.220, 0.677)	< 0.001	0.511 (0.241, 0.780)	< 0.001	− 0.072 (− 1.256, 1.111)	0.905	0.379
BMI-adjusted HC	39	211,088	4.953	596.237	− 0.122 (− 0.289, 0.046)	0.154	− 0.102 (− 0.347, 0.144)	0.417	0.305 (− 0.519, 1.130)	0.468	0.300
BMI-adjusted WC	43	211,088	5.565	611.484	0.136 (− 0.045, 0.317)	0.140	0.110 (− 0.115, 0.336)	0.338	0.610 (− 0.266, 1.486)	0.173	0.279
BMI-adjusted WHR	40	211,088	5.089	598.136	0.290 (0.101, 0.478)	0.003	0.310 (0.087, 0.532)	0.006	0.379 (− 0.636, 1.394)	0.464	0.861
BF percentage	41	89,297	5.207	597.795	0.475 (0.208, 0.741)	< 0.001	0.335 (− 0.008, 0.679)	0.055	0.028 (− 1.309, 1.365)	0.967	0.504
WLM	40	38,292	4.942	579.909	0.639 (− 0.579, 1.857)	0.304	0.831 (− 0.832, 2.495)	0.327	− 3.710 (− 10.054, 2.634)	0.252	0.171
ALM	40	28,330	4.942	579.909	− 0.233 (− 1.008, 0.542)	0.556	0.034 (− 1.017, 1.086)	0.949	− 3.363 (− 7.259, 0.533)	0.091	0.108
**Male-specific**											
BMI	38	152,882	4.731	583.146	0.417 (0.113, 0.720)	0.007	0.610 (0.289, 0.931)	< 0.001	0.378 (− 1.117, 1.874)	0.620	0.959
HC	37	93,480	4.522	571.095	0.197 (− 0.155, 0.549)	0.272	0.196 (− 0.193, 0.584)	0.323	1.451 (− 0.394, 3.297)	0.123	0.175
WC	35	93,480	4.299	572.741	0.476 (0.157, 0.796)	0.003	0.584 (0.175, 0.993)	0.005	0.646 (− 1.078, 2.370)	0.463	0.844
WHR	38	93,480	4.775	588.810	0.607 (0.315, 0.899)	< 0.001	0.399 (0.043, 0.755)	0.028	− 1.013 (− 2.499, 0.472)	0.181	0.029
BMI-adjusted HC	38	93,480	4.723	582.123	− 0.396 (− 0.699, − 0.092)	0.011	− 0.252 (− 0.625, 0.120)	0.184	0.727 (− 0.848, 2.303)	0.366	0.155
BMI-adjusted WC	40	93,480	5.133	603.571	0.097 (− 0.135, 0.329)	0.411	0.015 (− 0.313, 0.344)	0.927	0.802 (− 0.382, 1.987)	0.184	0.234
BMI-adjusted WHR	40	93,480	5.133	603.571	0.339 (0.114, 0.564)	0.003	0.230 (− 0.076, 0.536)	0.141	− 0.332 (− 1.489, 0.824)	0.574	0.246
BF percentage	41	44,429	5.207	597.795	0.617 (0.270, 0.963)	< 0.001	0.134 (− 0.322, 0.590)	0.564	− 0.410 (− 2.117, 1.298)	0.638	0.229
**Female-specific**											
BMI	36	171,963	4.452	577.461	0.571 (0.295, 0.848)	< 0.001	0.552 (0.243, 0.860)	< 0.001	0.011 (− 1.421, 1.443)	0.988	0.434
HC	37	116,742	4.669	590.581	0.455 (0.157, 0.752)	0.003	0.492 (0.168, 0.815)	0.003	0.747 (− 0.877, 2.371)	0.367	0.720
WC	33	116,742	4.249	600.039	0.480 (0.190, 0.769)	0.001	0.46 (0.125, 0.795)	0.007	− 0.030 (− 1.558, 1.498)	0.969	0.505
WHR	37	116,742	4.602	581.688	0.395 (0.115, 0.676)	0.006	0.501 (0.196, 0.805)	0.001	0.742 (− 0.825, 2.309)	0.353	0.659
BMI-adjusted HC	38	116,742	4.822	594.864	− 0.032 (− 0.239, 0.176)	0.766	− 0.096 (− 0.384, 0.192)	0.514	0.597 (− 0.488, 1.681)	0.281	0.248
BMI-adjusted WC	40	116,742	5.212	613.340	0.190 (− 0.027, 0.406)	0.085	0.288 (− 0.001, 0.576)	0.050	0.528 (− 0.585, 1.642)	0.352	0.544
BMI-adjusted WHR	38	116,742	4.659	573.819	0.249 (0.027, 0.472)	0.028	0.264 (− 0.028, 0.556)	0.076	1.057 (− 0.185, 2.298)	0.095	0.196
BF percentage	42	45,525	5.319	596.862	0.348 (0.027, 0.669)	0.034	0.414 (− 0.010, 0.838)	0.056	0.724 (− 0.882, 2.330)	0.377	0.639

*BMI* body mass index, *WC* waist circumference, *HC* hip circumference, *WHR* waist-to-hip ratio, *BF* body fat, *ALM* appendicular lean mass, *WLM* whole body lean mass, *SNP* single nucleotide polymorphism, *R*^*2*^* (%)* the percentage of exposure variance explained by SNPs, *95% CI* 95% confidence interval.

### Univariable MR

**Figure 1:**
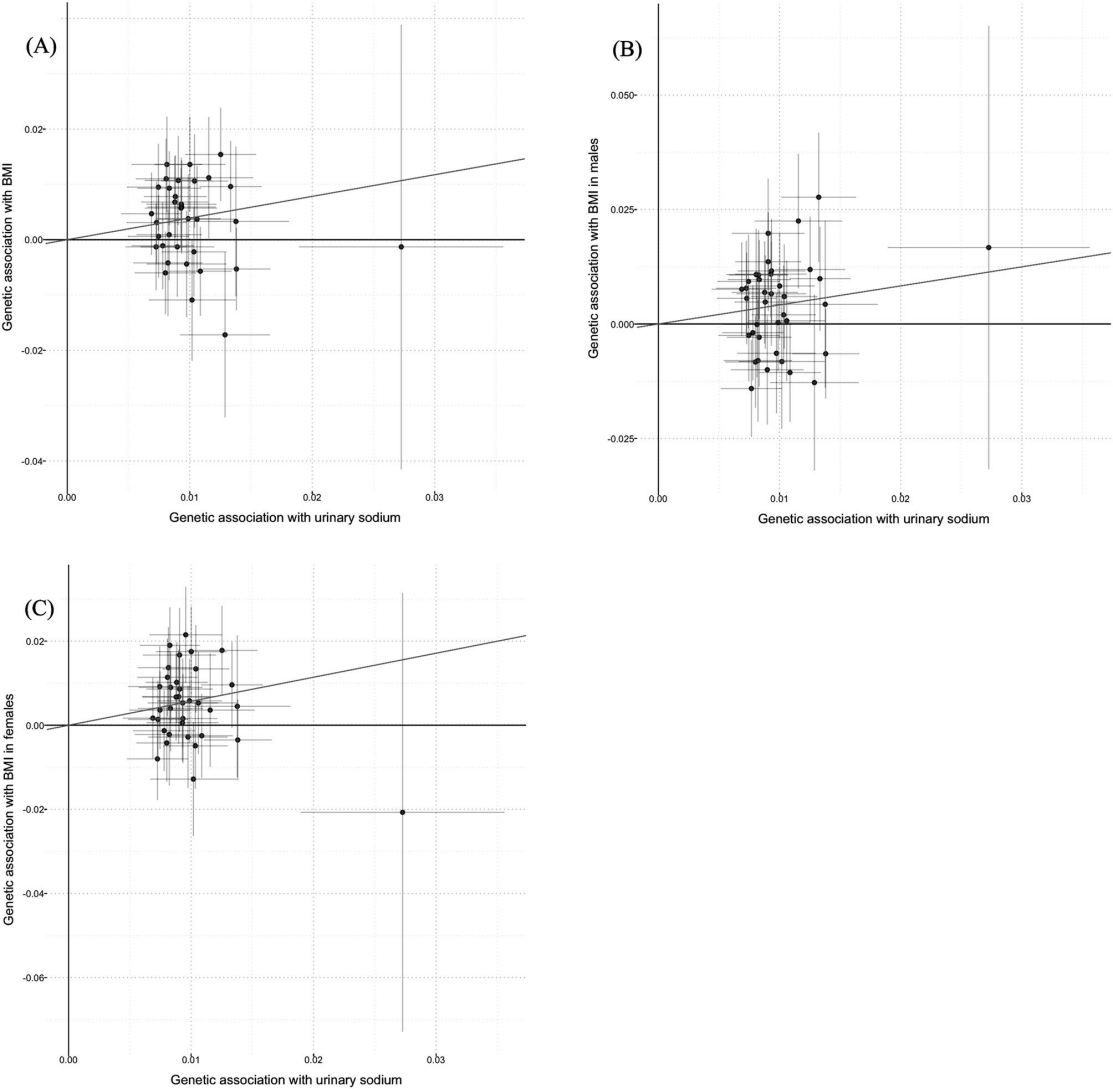
Scatter plots for Mendelian randomization analysis of urinary sodium and BMI. (**A**) sex-combined, (**B**) male-specific, (**C**) female-specific. Horizontal axis: SNPs’ association with urinary sodium. Vertical axis: SNPs’ association with BMI. The fitted line showed the results of the inverse-variance weighted method in univariable MR.

**Figure 2:**
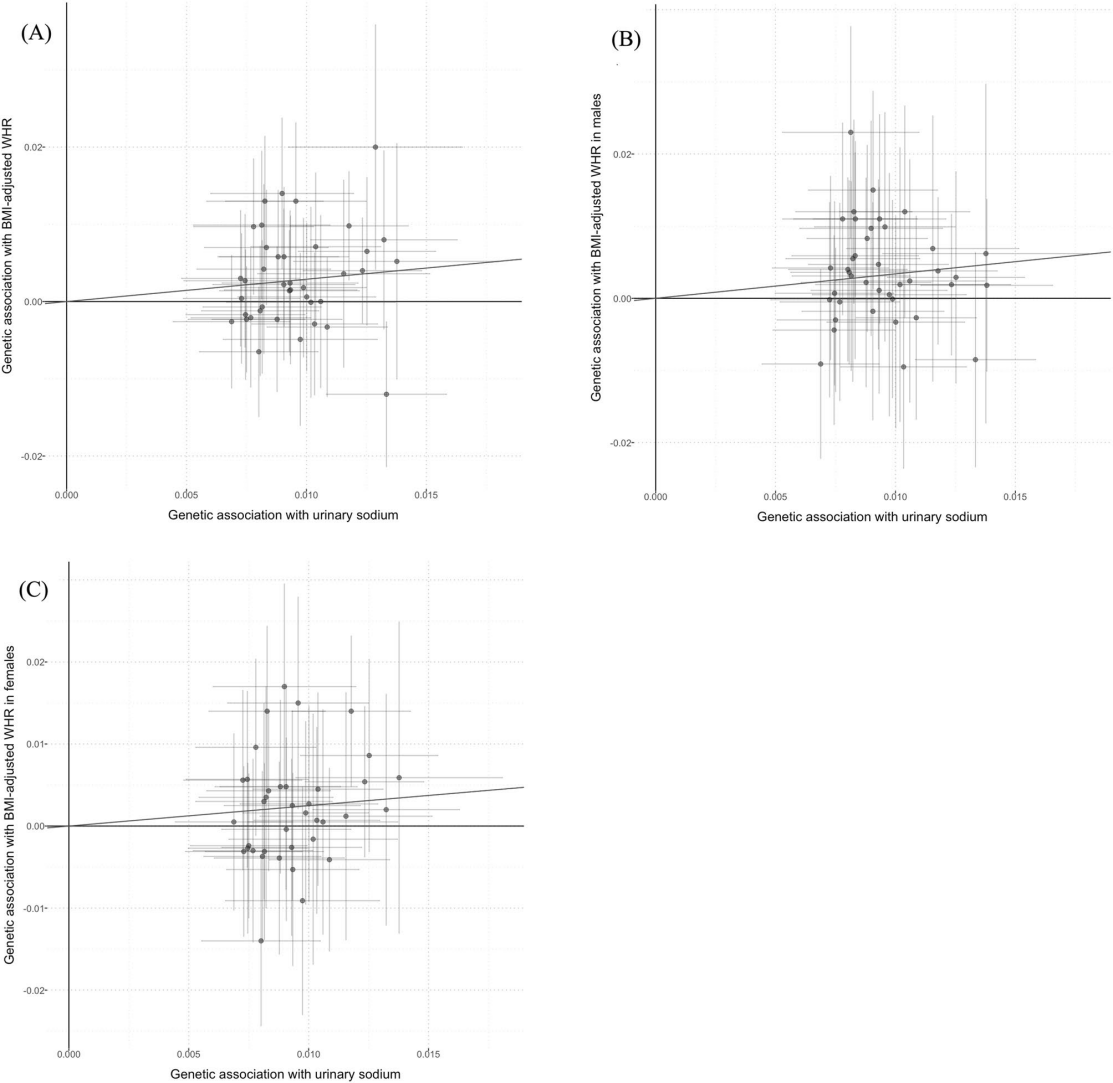
Scatter plots for Mendelian randomization analysis of urinary sodium and BMI-adjusted waist-to-hip ratio. (**A**) Sex-combined, (**B**) male-specific, (**C**) female-specific. Horizontal axis: SNPs’ association with urinary sodium. Vertical axis: SNPs’ association with BMI-adjusted waist-to-hip ratio. The fitted line showed the results of the inverse-variance weighted method in univariable MR.

**Figure 3:**
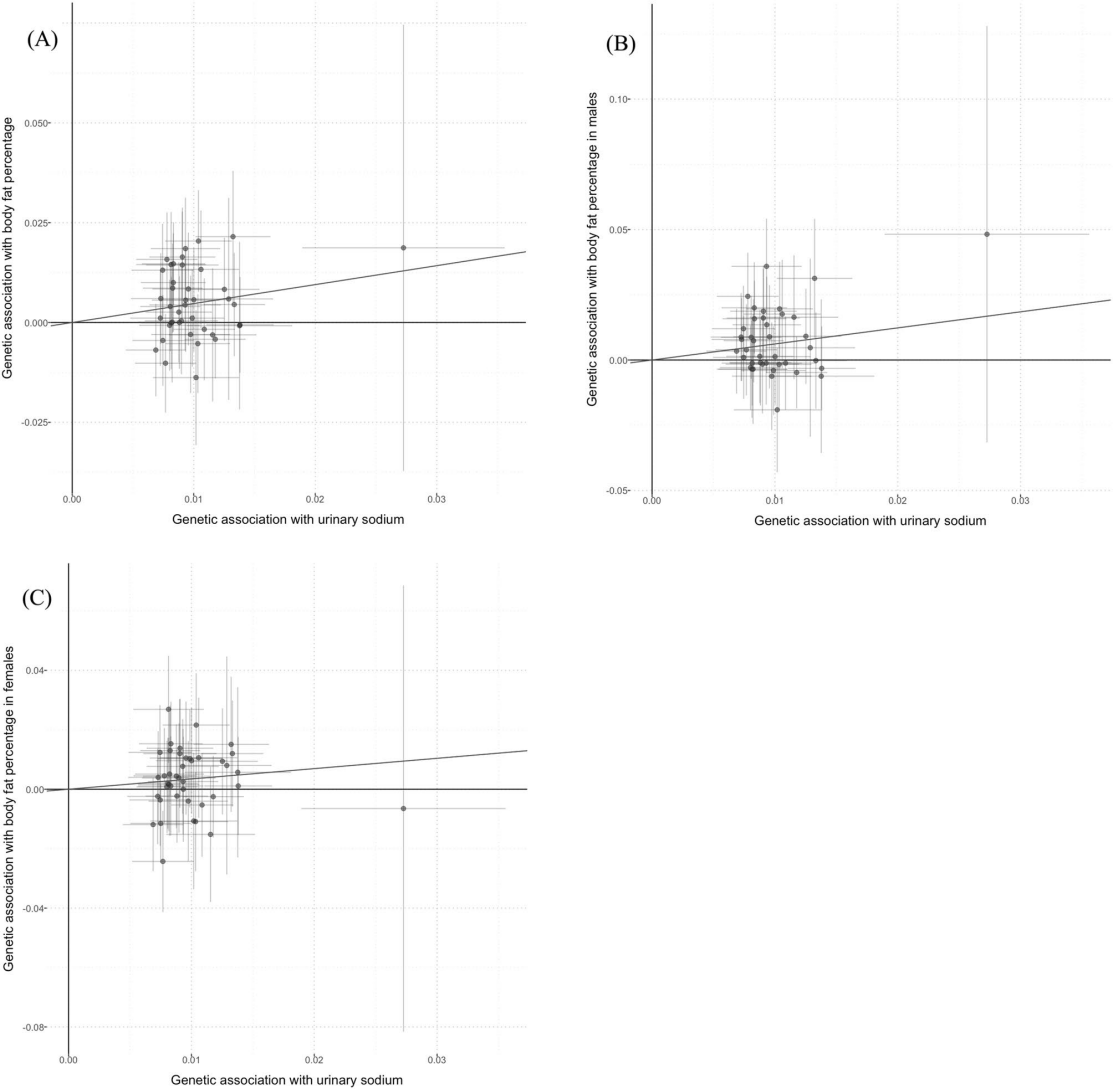
Scatter plots for Mendelian randomization analysis of urinary sodium and body fat percentage. (**A**) Sex-combined, (**B**) male-specific, (**C**) female-specific. Horizontal axis: SNPs’ association with urinary sodium. Vertical axis: SNPs’ association with body fat percentage. The fitted line showed the results of the inverse-variance weighted method in univariable MR.

In sex-combined model, univariate MR analyses demonstrated positive causal associations between UNa with BMI (Fig. 1), BMI-adjusted WHR (Fig. 2) and BF percentage (Fig. 3) in both sex-combined and sex-specific models (Table 1). MR-Egger intercept tests revealed no horizontal pleiotropy for these outcomes. BMI was positively related to UNa [beta (95% CI): 0.042 (0.022–0.062)], which suggested a bidirectional association between UNa and BMI.

### Multivariable MR

UNa showed independent positive association with BMI [0.406 (0.202–0.610)], BMI-adjusted WHR [0.222 (0.029–0.416)] and BF percentage [0.471 (0.198–0.744)], after adjusted for eGFR. No association was observed with BMI-adjusted HC, BMI-adjusted WC, WLM or ALM. MR-Egger intercept tests revealed no pleiotropy (Table 2). The bidirectional association between BMI and UNa remained after adjusted for eGFR. The eGFR-adjusted effect of BMI on UNa was 0.043 (0.023–0.063) with no pleiotropy (P for MR-Egger intercept test = 0.149).

**Table 2:** Results of multivariable Mendelian randomization analysis (inverse-variance weighted method) adjusted for estimated glomerular filtration rate.

Outcome	SNPs	N	R^2^ (%)	F statistic	Inverse-variance weighted method		MR-Egger method			P for heterogeneity between sexes
					Beta (95% CI)	P for beta	Beta (95% CI)	P for beta	P for Egger intercept	
**Sex-combined**										
BMI	34	322,154	4.227	579.157	0.406 (0.202, 0.610)	< 0.001	0.319 (− 0.736, 1.373)	0.554	0.868	0.439
HC	35	211,088	4.036	536.146	0.299 (0.055, 0.544)	0.017	0.449 (− 0.998, 1.896)	0.543	0.837	0.768
WC	37	211,088	4.546	574.298	0.533 (0.283, 0.783)	< 0.001	0.486 (− 0.821, 1.794)	0.466	0.943	0.359
WHR	38	211,088	4.703	579.443	0.385 (0.145, 0.624)	0.002	− 0.371 (− 1.567, 0.825)	0.543	0.207	0.225
BMI-adjusted HC	39	211,088	4.953	596.237	− 0.094 (− 0.259, 0.070)	0.261	0.277 (− 0.523, 1.078)	0.497	0.353	0.073
BMI-adjusted WC	43	211,088	5.565	611.484	0.121 (− 0.045, 0.287)	0.154	0.719 (− 0.078, 1.516)	0.077	0.133	0.723
BMI-adjusted WHR	40	211,088	5.089	598.136	0.222 (0.029, 0.416)	0.025	0.052 (− 0.979, 1.083)	0.921	0.742	0.351
BF percentage	41	89,297	5.207	597.795	0.471 (0.198, 0.744)	0.001	0.024 (− 1.331, 1.379)	0.972	0.509	0.242
WLM	40	38,292	4.942	579.909	0.633 (− 0.587, 1.853)	0.309	− 3.705 (− 10.074, 2.665)	0.254	0.174	NA
ALM	40	28,330	4.942	579.909	− 0.246 (− 1.029, 0.536)	0.537	− 3.276 (− 7.231, 0.679)	0.104	0.126	NA
**Male-specific**										
BMI	38	152,882	4.731	583.146	0.443 (0.163, 0.724)	0.002	0.755 (− 0.650, 2.159)	0.292	0.657	
HC	37	93,480	4.522	571.095	0.228 (− 0.102, 0.557)	0.176	2.204 (0.492, 3.915)	0.012	0.021	
WC	35	93,480	4.299	572.741	0.526 (0.238, 0.813)	< 0.001	1.335 (− 0.241, 2.911)	0.097	0.306	
WHR	38	93,480	4.775	588.810	0.598 (0.300, 0.896)	< 0.001	− 1.020 (− 2.522, 0.482)	0.183	0.032	
BMI-adjusted HC	38	93,480	4.723	582.123	− 0.368 (− 0.673, − 0.063)	0.018	0.693 (− 0.879, 2.266)	0.388	0.178	
BMI-adjusted WC	40	93,480	5.133	603.571	0.079 (− 0.156, 0.314)	0.508	0.784 (− 0.402, 1.969)	0.195	0.235	
BMI-adjusted WHR	40	93,480	5.133	603.571	0.321 (0.094, 0.548)	0.006	− 0.346 (− 1.503, 0.811)	0.557	0.249	
BF percentage	41	44,429	5.207	597.795	0.622 (0.268, 0.976)	0.001	− 0.406 (− 2.135, 1.323)	0.645	0.234	
**Female-specific**										
BMI	36	171,963	4.452	577.461	0.594 (0.333, 0.855)	< 0.001	0.361 (− 1.034, 1.755)	0.612	0.739	
HC	37	116,742	4.669	590.581	0.437 (0.135, 0.739)	0.005	0.710 (− 0.926, 2.345)	0.395	0.740	
WC	33	116,742	4.249	600.039	0.464 (0.170, 0.758)	0.002	− 0.053 (− 1.592, 1.485)	0.946	0.502	
WHR	37	116,742	4.602	581.688	0.337 (0.039, 0.636)	0.027	0.471 (− 1.184, 2.125)	0.577	0.872	
BMI-adjusted HC	38	116,742	4.822	594.864	− 0.028 (− 0.240, 0.184)	0.796	0.598 (− 0.501, 1.697)	0.286	0.256	
BMI-adjusted WC	40	116,742	5.212	613.340	0.135 (− 0.067, 0.336)	0.190	0.430 (− 0.594, 1.453)	0.411	0.564	
BMI-adjusted WHR	38	116,742	4.659	573.819	0.170 (− 0.052, 0.391)	0.133	0.702 (− 0.534, 1.938)	0.266	0.391	
BF percentage	42	45,525	5.319	596.862	0.334 (0.007, 0.662)	0.045	0.713 (− 0.908, 2.334)	0.388	0.640	

*BMI* body mass index, *WC* waist circumference, *HC* hip circumference, *WHR* waist-to-hip ratio, *BF* body fat, *ALM* appendicular lean mass, *WLM* whole body lean mass, *SNP* single nucleotide polymorphism, *R*^*y*^*(%)* the percentage of exposure variance explained by SNPs, *95% CI* 95% confidence interval, *NA* not applicable.

For men, UNa was positively associated with BMI [eGFR-adjusted beta 0.443 (0.163–0.724)], BMI-adjusted WHR [0.321 (0.094–0.548)], and BF percentage [0.622 (0.268–0.976)], but negatively with BMI-adjusted HC [− 0.368 (− 0.673 to − 0.063)]. No pleiotropy was observed for these outcomes, although pleiotropy was observed in secondary outcomes, such as HC and WHR (Table 2).

For women, UNa was associated with BMI [eGFR-adjusted beta 0.594 (0.333–0.855)] and BF percentage [0.334 (0.007–0.662)], but not BMI-adjusted WHR [0.170 (− 0.052 to 0.391)]. No pleiotropy was observed (Table 2).

Overall, UNa was associated with increased BMI and BF percentage in both men and women. Higher UNa affected male body shape by increasing BMI-adjusted WHR and decreasing BMI-adjusted HC. UNa seemed to have little effects on female body shape after adjusted for BMI and eGFR. Formal heterogeneity test showed significant difference between men and women on BMI-adjusted HC (Table 2).

In sensitivity analysis including the SNPs that were identified as outliers by MR-PRESSO outlier tests, both the univariable and multivariable MR showed results consistent with primary results (Supplement Tables S2, S3). Removing the eGFR-associated SNP rs1260326 also generated concordant results (Supplementary Table S4).

The scatter plots showed a potential outlier SNP rs4803378, located in gene *CTC-490E21.10* and associated with urine creatinine according to PhenoScanner v2 database, the post hoc sensitivity analyses by removing this SNP showed similar results to the primary results (Supplementary Table S5).

## Discussion

UNa was causally associated with increased BMI and BF percentage. Higher UNa changed body shape towards central obesity in men, by reducing BMI-adjusted HC and increasing BMI-adjusted WHR, but probably not in women. There was a bidirectional positive association between UNa and BMI.

UNa has been widely used as a surrogate for dietary salt intake^9,10,37^. UNa is mainly determined by dietary sodium intake and kidney function, and 24-h UNa is favored than spot UNa. Some validated equations were used to estimate 24-h urinary sodium from spot urinary sodium, which additionally considers urinary creatinine or kidney function, such as Kawasaki equation, INTERSALT equation^38–40^. Spot urine sample was collected in UK Biobank^24^. Although we did not estimate 24-h UNa due to lack of individual data in this study, which is one of the limitations, we adjusted for kidney function (eGFR) in multivariable analysis, which enhanced the reliability of our estimation of the effect size of dietary salt intake on the outcomes. Nevertheless, future studies using individual 24-h UNa or dietary sodium intake are warranted.

These results were consistent with previous findings. Moosavian et al.^13^ found in a meta-analysis that both dietary salt intake and UNa were positively associated with BMI. This study computed the effect measure as the BMI mean difference between highest and lowest categories of exposure, which introduced heterogeneity due to various cutoff values used in included studies and made it difficult to directly compare the effect size with other studies. Zhou et al.^14^ found that for each 1 gram per day (g/day) increase in salt intake, BMI increased by 0.42 and 0.52 kg/m^2^, overweight/obesity risk increased by 29% and 24% in UK and US population, respectively, after adjustment for total energy intake and physical activity.

UNa showed an effect on body composition by increasing BF percentage, which is consistent with previous observational findings. An increase of 1 g/d salt intake was associated with an increase of 0.91 kg BF mass in the UK population^21^, and 0.79 kg BF mass and 0.44% BF percentage in US population^17^. Oh et al.^41^ observed this positive association but only in people under 65 years old. Zhu et al.^42^ found salt intake was associated with increased BF mass, BF percentage, and subcutaneous abdominal adipose tissue. Interestingly, a randomized trial^20^ found that low-salt diet intervention significantly decreased body weight, BMI and extracellular water, but reductions in BF mass and lean mass were insignificant. However, this trial was limited by small sample size (84 obese people) and short follow-up period (2 months), which might make it underpowered to detect significant reductions. Salt intake has an instant effect on water retention, but its effect on fat accumulation may take longer.

We failed to find the effect of UNa on lean mass, neither WLM nor ALM. This remains uncertain. Previously, Ma et al.^15^ showed that 1 g/d increase in salt intake was associated with significant WLM increase by 0.32 kg, but 1 g/2000 kcal increase in salt intake density insignificantly reduced WLM by 0.007 kg. Zhang et al.^17^ found that salt intake significantly increased WLM by 0.53 kg, but the effect became insignificant when using salt intake density (g/kcal) to adjust for energy intake. It seems that there are two possible effects of salt intake on body composition: (1) increase both fat and lean mass, but fat mass increases at a higher rate; (2) increase fat mass solely. The first explanation was supported by the findings that salt intake was associated with more BF mass than lean mass in both UK (0.91 kg versus 0.32 kg for fat and lean mass increase, respectively)^15^ and US populations (0.79 kg versus 0.53 kg)^17^. The second explanation was supported by the the null effect of salt intake on lean mass after adjustment for energy intake^15,17^. Our findings also support the second explanation. Nevertheless, more research is warranted on this topic.

There are several potential explanations for how salt intake affects body weight and composition. It has been well recognized that excessive salt intake increases thirst and fluid consumption, thus increasing extracellular water while keeping urine volume almost unchanged^43^. Higher dietary salt intake also increases sweetened-beverage intake^44^, food appetite, food consumption and calorie intake^13,45^, which all contribute to weight gain. More importantly, high sodium intake has independent biological effects on fat accumulation. Lanaspa et al*.*^46^ demonstrated that high salt intake activated the aldose reductase-fructokinase pathway in liver and hypothalamus, causing endogenous fructose production and the development of leptin resistance and hyperphagia, which eventually cause obesity. Fonseca-Alaniz et al*.*^47^ observed that the high salt-induced adiposity in rats was characterized by high plasma leptin concentrations and adipocyte hypertrophy, which might be mediated by lipogenic capacity of white adipose mass. Lee et al*.*^48^ revealed that high salt increased the expression of adipogenic and lipogenic genes (such as *PPAR-γ, SREBP1c, ACC, C/EBPα,* and *FAS*), but decreased lipolysis gene expression (such as *AMPK*). However, its effect on lean mass requires more replications and mechanism research.

We found that UNa had an impact on body shape. Body shape, rather than simple BMI, is more predictive of obesity-related mortality and health outcomes^49,50^. Overall, UNa increased both BMI-adjusted and unadjusted WHR, leading to central obesity, which is in line with previous findings^13,16,17,42^. However, we observed an effect modification caused by sex. UNa increased WHR in both sexes, but a significant increase in BMI-adjusted WHR was seen in men only. Nam et al*.*^16^ observed a similar sex difference. This may suggest that UNa affects body shape independent of BMI in men, but its effect on female body shape is, at least partly, mediated by BMI.

This sexual dimorphism of body fat accumulation and distribution may be related to sex hormones^51,52^, sex chromosome^53^ and sex-specific autosomal genetic heritability^22,23,54,55^. Estrogens favor fat accumulation in subcutaneous depot in women. Estrogens and their receptor regulations also enhance the expandability of adipose cells in subcutaneous depot and inhibits it in visceral depots^51^. Elevated free androgens have also been shown to be related to increased abdominal visceral fat accumulation and increased WC^56^. The Four Core Genotypes mouse model studies, have shown that X chromosome dosage is a risk factor for fat accumulation and obesity, but the presence of Y chromosome is not^54^. Other studies have also shown that that adipocytes from different subcutaneous depots (abdominal versus gluteal) are developmentally distinct in a sex-dependent manner^57,58^. The GWA study of BMI-adjusted WHR showed a higher heritability in women than in men (2.4% versus 0.8%)^26^. This indicates that male body shape is more likely to be influenced by environmental factors^54^, such as salt intake. However, mechanistic studies are needed to elucidate this issue.

In this MR study, we observed that high BMI was causally linked to higher UNa. High UNa could be resulted from two possible mechanisms: obesity-related glomerulopathy and high salt intake. Obesity is known to increase risk of glomerulopathy, characterized with altered renal hemodynamics, increased eGFR, filtration fraction, tubular sodium reabsorption, glomerular hyperfiltration, hormonal and neurohormonal activation^59^; however, the overall effect of glomerulopathy on UNa remains unclear. On the other hand, the association between BMI and UNa remained consistent after adjusting for renal function (eGFR) in multivariable MR, therefore, we believe that salt intake has independent mediation effect on the association between BMI and UNa, that is, BMI increases UNa by increasing salt intake, independent of renal function. Li et al.^60^ found that overweight/obese people had lower salt sensitivity and higher salt preference, and consumed 2.3 g/day more dietary salt than normal-weight people.

The bidirectional association between obesity and salt intake indicates the formation of a vicious cycle, in which people with higher BMI are prone to consume more salt, which then further increases BMI. Considering that high salt intake and obesity, both prevalent worldwide, are shared risk factors for many chronic conditions and diseases, and that they also have their own adverse health consequences and disease burdens^61^, urgent interventions are required to break this cycle in high-risk individuals.

Salt reduction and weight reduction programs have been being widely implemented in various settings, but separately. Salt reduction programs do not consider weight change as an objective, while weight reduction programs pay little emphasis on salt reduction measures. We are not advocating weight control by only restricting salt. Instead, our findings provide evidence to combine salt reduction and weight reduction measures, to facilitate people to acquire a healthier lifestyle and achieve higher health benefits. Although the actual effect size of salt reduction on weight reduction has not been precisely estimated yet, which warrants future well-designed randomized trials, salt reduction per se costs less and brings additional health benefits^18,41,62^.

There are some limitations. First, we did not include all identified UNa-associated SNPs due to unmatched data and pleiotropy issues; however, our instrument variables were strong (high F statistics) and the results are consistent in primary analyses, sensitivity analyses, and with previous studies. Second, more accurate UNa measure is via 24-h urine sample collection, but the UK Biobank used spot urine sample. 24-h urine collection is more expensive, time-consuming, labor-intensive and difficult for an epidemiological study as large as UK Biobank with half a million participants. We adjusted for kidney function in multivariable MR, which is considered in previous validated equations converting spot UNa to 24-h UNa^38,39^. Nevertheless, future studies with 24-h UNa measurement or dietary salt intake are warranted. Third, there might still be residual pleiotropy, although we have removed SNPs that were significantly associated with outcomes and that were identified as outliers by the MR-PRESSO outlier test. MR-Egger intercept test showed pleiotropy for a few secondary outcomes (e.g., HC, WC), but not for the primary outcomes. Fourth, the effect size estimated in this study was not directly comparable to other studies in which the effect was measured at natural units of outcome by 1 g/day salt intake increase. Although several equations have been proposed to estimate salt intake in g/day from 24 h urine sodium secretion in g/d or spot urine sodium concentration in mmol/L^63^, the UNa GWA study log-transformed the original spot UNa concentration, which makes back-transformation of beta estimate to natural unit impossible. Due to the same reason, formal power calculations were not performed as we planned, which was another limitation, but our study has employed strong instrumental variables and so far the largest GWA studies available. Lastly, we only included European-ancestry population, thus generalizing the results to other ethnicities requires cautions.

## Conclusion

UNa increased BMI and BF percentage, and changes male body shape towards central obesity by increasing BMI-adjusted WHR. Given the bidirectional association between salt intake and obesity, salt reduction measures and weight reduction measures should be implemented together to achieve more health benefits.

## Supplementary information

Supplementary Information 1

Supplementary Information 2

## Data Availability

All data and analytic code are available upon request.
