# Supplementary material for: Causal associations between urinary sodium with body mass, shape and composition: a Mendelian randomization study
Source: Sci Rep. 2020 Oct 15;10:17475. doi: 10.1038/s41598-020-74657-x (PMC7562909; doi:10.1038/s41598-020-74657-x)
Supplement: Supplementary file 1 — Supplementary Information 1 [file 41598_2020_74657_MOESM1_ESM.docx]

Causal associations between urinary sodium with body mass, shape and composition: a Mendelian randomization study

Feng Q, et al.

**Supplementary file 1**. Characteristics of relevant SNPs and their associations with the exposure and the outcomes in Mendelian randomization analysis

The file is an Excel sheetbook and available at the following link:

<https://www.medrxiv.org/highwire/filestream/84249/field_highwire_adjunct_files/0/2020.05.01.20087007-1.xlsx>
