# Supplementary material for: Causal associations between urinary sodium with body mass, shape and composition: a Mendelian randomization study
Source: Sci Rep. 2020 Oct 15;10:17475. doi: 10.1038/s41598-020-74657-x (PMC7562909; doi:10.1038/s41598-020-74657-x)
Supplement: Supplementary file 2 — Supplementary Information 2 [file 41598_2020_74657_MOESM2_ESM.docx]

Causal associations between urinary sodium with body mass, shape and composition: a Mendelian randomization study

Feng Q, et al.

Supplementary file 2. Characteristics of the 50 SNPs and the results of sensitivity analyses

Elaboration regarding included SNPs and MR analysis methods for the tables presented in the manuscript and this supplementary file 2

| SNPs | SNP criteria | Univariate MR | Multivariate MR |
| --- | --- | --- | --- |
| (1) | 50 SNPs originally identified in the UNa GWA study | -- | -- |
| (2) | (1) - SNPs associated with the outcomes at significance level of 5*10^-8^ | Supplementary table 2 | Supplementary table 3 |
| (3) | (2) - SNP outliers identified by MR-PRESSO outlier test | Table 1 | Table 2 |
| (4) | (3) - SNP rs1260362, which is associated with eGFR | Supplementary table 4 | -- |
| (5) | (4) - SNP rs4803378, the outlier in scatter plots | Supplementary table 5 | -- |

Supplementary table 1. Basic characteristics of urinary sodium secretion-associated SNPs and their association with urinary sodium secretion.

| SNP | Proxy SNP | Gene | Proxy information | Chromosome:  position | Effect allele | Other allele | Effect allele frequency | beta | Standard error | p value |
| --- | --- | --- | --- | --- | --- | --- | --- | --- | --- | --- |
| rs10136360 | rs10150482 | NRXN3 | R^2^=1.00, D=1.00 | 14:79891882 | A | G | 0.221 | 0.010 | 0.001 | 1.70E-10 |
| rs1036336 | rs1036336 | PLCL1 | NA | 2:198964120 | T | C | 0.489 | 0.007 | 0.001 | 3.90E-09 |
| rs10457480 | rs10457480 | MIR588 | NA | 6:126812609 | G | C | 0.302 | 0.008 | 0.001 | 6.80E-10 |
| rs11584700 | rs11584700 | LRRN2 | NA | 1:204576983 | A | G | 0.791 | 0.009 | 0.002 | 3.40E-09 |
| rs11642015 | rs1421085 | FTO | R^2^=1.00, D=1.00 | 16:53800954 | C | T | 0.404 | 0.012 | 0.001 | 1.30E-22 |
| rs11646118 | rs11646118 | KCTD13 | NA | 16:29926471 | T | C | 0.913 | 0.014 | 0.002 | 3.70E-10 |
| rs117287096 | rs4803378 | CYP2A6 | R^2^=0.87, D=1.00 | 19:41333441 | A | G | 0.022 | 0.027 | 0.004 | 1.20E-10 |
| rs1229984 | NA | ADH1B | No proxy at R^2^=0.80 | 4:100239319 | T | C | 0.027 | 0.024 | 0.004 | 2.00E-10 |
| rs12581220 | rs12581220 | LINC02424 | NA | 12:78773251 | C | T | 0.686 | 0.010 | 0.001 | 1.20E-14 |
| rs1260326 | rs1260326 | GCKR | NA | 2:27730940 | T | C | 0.396 | 0.012 | 0.001 | 1.00E-20 |
| rs13163314 | rs13188076 | HTR4 | R^2^=1.00, D=1.00 | 5:148099993 | G | T | 0.768 | 0.010 | 0.001 | 8.80E-12 |
| rs141086701 | NA | -- | No proxy at R^2^=0.80 | 8:143361458 | G | A | 0.992 | 0.045 | 0.007 | 9.30E-10 |
| rs1437971 | rs7562372 | CHST10 | R^2^=0.89, D=0.98 | 2:100906331 | T | C | 0.350 | 0.007 | 0.001 | 1.10E-08 |
| rs1513481 | NA | MIR128-2 | No proxy at R^2^=0.80 | 3:35783535 | T | C | 0.311 | 0.009 | 0.001 | 3.30E-12 |
| rs1516187 | rs1516187 | LOC730100 | NA | 2:51930164 | C | T | 0.868 | 0.012 | 0.002 | 3.00E-10 |
| rs1562308 | rs1562308 | XPO1 | NA | 2:61768421 | C | A | 0.604 | 0.008 | 0.001 | 2.20E-10 |
| rs16850592 | rs16850592 | SCN2A | NA | 2:166265016 | T | C | 0.759 | 0.009 | 0.001 | 1.30E-10 |
| rs17123039 | rs17123039 | ABHD12B | NA | 14:51332335 | T | G | 0.582 | 0.007 | 0.001 | 7.70E-09 |
| rs17635778 | rs17635778 | BRINP1 | NA | 9:122165102 | C | T | 0.826 | 0.010 | 0.002 | 2.80E-09 |
| rs1957111 | NA | PRKD1 | No proxy at R^2^=0.80 | 14:29781178 | T | C | 0.052 | 0.019 | 0.003 | 1.00E-11 |
| rs1996928 | rs6126631 | LINC01525 | R^2^=0.90, D=1.00 | 20:51255210 | A | C | 0.761 | 0.008 | 0.001 | 2.20E-08 |
| rs2393831 | rs2393831 | CABCOCO1 | NA | 10:63466754 | A | G | 0.777 | 0.009 | 0.001 | 4.40E-10 |
| rs2422137 | rs2422137 | NEGR1 | NA | 1:72619961 | C | A | 0.546 | 0.008 | 0.001 | 1.60E-09 |
| rs2472297 | rs2472297 | CYP1A1 | NA | 15:75027880 | C | T | 0.737 | 0.014 | 0.001 | 6.70E-23 |
| rs2504671 | rs2504671 | PKHD1 | NA | 6:51205577 | C | A | 0.765 | 0.013 | 0.001 | 1.00E-17 |
| rs2761589 | rs2210144 | DCDC1 | R^2^=0.98, D=1.00 | 11:31253268 | G | T | 0.633 | 0.009 | 0.001 | 6.10E-12 |
| rs2945091 | rs2959129 | -- | R^2^=1.00, D=1.00 | 11:28851988 | C | G | 0.439 | 0.007 | 0.001 | 3.40E-08 |
| rs2968426 | rs2968426 | MIR5093 | NA | 16:85335735 | G | C | 0.255 | 0.008 | 0.001 | 7.90E-09 |
| rs33951980 | rs35332062 | MLXIPL | R^2^=0.92, D=1.00 | 7:73012042 | G | A | 0.872 | 0.013 | 0.002 | 3.50E-12 |
| rs34452566 | NA | BDNF | No proxy at R^2^=0.80 | 11:27793470 | T | G | 0.199 | 0.010 | 0.002 | 3.30E-10 |
| rs34783010 | rs10423928 | MIR642A | R^2^=1.00, D=1.00 | 19:46182304 | T | A | 0.806 | 0.013 | 0.002 | 2.20E-17 |
| rs40465 | rs40465 | RAB9BP1 | NA | 5:103981726 | G | T | 0.332 | 0.008 | 0.001 | 2.50E-10 |
| rs4410790 | rs4410790 | AHR | NA | 7:17284577 | T | C | 0.369 | 0.011 | 0.001 | 2.30E-17 |
| rs4442732 | rs4442732 | SIX6 | NA | 14:61025617 | G | A | 0.623 | 0.008 | 0.001 | 1.00E-09 |
| rs4788415 | NA | DCTPP1 | No proxy at R^2^=0.80 | 16:30439309 | C | T | 0.903 | 0.015 | 0.002 | 4.60E-12 |
| rs55807911 | NA | LOC100505736 | No proxy at R^2^=0.80 | 2:28421473 | C | T | 0.198 | 0.010 | 0.002 | 4.80E-10 |
| rs5760425 | rs5760425 | SPECC1L-ADORA2A | NA | 22:24842452 | T | G | 0.391 | 0.007 | 0.001 | 3.90E-09 |
| rs643428 | rs630602 | SSBP3-AS1 | R^2^=1.00, D=1.00 | 1:54728864 | G | C | 0.393 | 0.008 | 0.001 | 1.60E-09 |
| rs7072776 | rs7072776 | LINC01114 | NA | 10:22032942 | A | G | 0.278 | 0.010 | 0.001 | 6.50E-14 |
| rs7140993 | rs7140993 | MLLT10 | NA | 14:27107166 | G | A | 0.738 | 0.009 | 0.001 | 3.60E-11 |
| rs72634682 | Rs6468262 | NOVA1-AS1 | R^2^=1.00, D=1.00 | 8:34352645 | C | A | 0.138 | 0.010 | 0.002 | 1.40E-08 |
| rs7334078 | rs7334078 | LINC01288 | NA | 13:99120484 | T | C | 0.717 | 0.009 | 0.001 | 4.30E-11 |
| rs7442885 | rs7442885 | STK24 TMEM161B- | NA | 5:87682877 | C | G | 0.787 | 0.009 | 0.002 | 2.10E-09 |
| rs7619139 | rs7619139 | AS1 | NA | 3:25110415 | A | T | 0.589 | 0.008 | 0.001 | 9.60E-11 |
| rs784257 | rs784257 | RARB | NA | 18:53397199 | C | T | 0.812 | 0.011 | 0.002 | 2.90E-11 |
| rs7924036 | rs7070761 | LINC01415 | R^2^=0.96, D=1.00 | 10:65317056 | T | A | 0.486 | 0.008 | 0.001 | 2.40E-11 |
| rs816366 | rs816366 | LOC105378330 | NA | 6:53995504 | C | T | 0.311 | 0.010 | 0.001 | 1.40E-13 |
| rs838133 | rs838133 | MLIP | NA | 19:49259529 | G | A | 0.549 | 0.013 | 0.001 | 1.90E-25 |
| rs9387963 | rs9372650 | FGF21 | R^2^=1.00, D=1.00 | 6:98395421 | A | C | 0.557 | 0.008 | 0.001 | 1.00E-10 |
| rs9537160 | rs9537160 | MIR2113 | NA | 13:56109838 | C | T | 0.722 | 0.009 | 0.001 | 2.30E-10 |

SNP: single nucleotide polymorphism. NA: not applicable. Chromosome:position was based on GRCh37.

Supplementary table 2. Results of univariable Mendelian randomization analyses (showing beta (95% confidence interval)) of sex-combined and sex-specific association between urinary sodium secretion with body mass, shape and composition outcomes, using all relevant SNPs^†^

| Outcome | SNP | Inverse-variance weighted method | Weighted median method | MR-Egger method | P for Egger intercept |
| --- | --- | --- | --- | --- | --- |
| **Sex-combined** |  |  |  |  |  |
| BMI | 40 | 0.405 (0.107, 0.703) | 0.544 (0.275, 0.814) | -0.691 (-2.178, 0.795) | 0.140 |
| HC | 42 | 0.242 (-0.090, 0.573) | 0.240 (-0.047, 0.527) | 1.135 (-0.501, 2.771) | 0.274 |
| WC | 40 | 0.447 (0.152, 0.742) | 0.430 (0.144, 0.716) | 0.160 (-1.301, 1.620) | 0.694 |
| WHR | 42 | 0.398 (0.128, 0.667) | 0.397 (0.138, 0.656) | -0.220 (-1.547, 1.107) | 0.351 |
| BMI-adjusted HC | 42 | -0.135 (-0.375, 0.105) | -0.099 (-0.340, 0.143) | 1.105 (-0.013, 2.223) | 0.026 |
| BMI-adjusted WC | 43 | 0.136 (-0.045, 0.317) | 0.110 (-0.115, 0.336) | 0.610 (-0.266, 1.486) | 0.279 |
| BMI-adjusted WHR | 43 | 0.243 (0.034, 0.453) | 0.254 (0.010, 0.497) | -0.451 (-1.464, 0.562) | 0.170 |
| BF percentage | 42 | 0.408 (0.121, 0.695) | 0.319 (-0.006, 0.643) | 0.437 (-1.002, 1.876) | 0.968 |
| WLM | 42 | 0.642 (-0.847, 2.132) | 0.832 (-0.939, 2.603) | -2.621 (-10.212, 4.969) | 0.390 |
| ALM | 42 | -0.165 (-1.087, 0.756) | 0.038 (-1.057, 1.134) | -2.339 (-6.973, 2.294) | 0.348 |
|  |  |  |  |  |  |
| **Male-specific** |  |  |  |  |  |
| BMI | 41 | 0.401 (0.050, 0.752) | 0.586 (0.255, 0.918) | -0.402 (-2.139, 1.334) | 0.355 |
| HC | 39 | 0.089 (-0.304, 0.481) | 0.110 (-0.294, 0.515) | 0.417 (-1.681, 2.514) | 0.755 |
| WC | 39 | 0.333 (-0.084, 0.749) | 0.478 (0.065, 0.892) | 0.309 (-1.924, 2.542) | 0.983 |
| WHR | 39 | 0.520 (0.198, 0.842) | 0.375 (-0.002, 0.753) | -0.357 (-2.059, 1.345) | 0.304 |
| BMI-adjusted HC | 40 | -0.373 (-0.717, -0.029) | -0.227 (-0.606, 0.152) | 1.516 (-0.170, 3.202) | 0.025 |
| BMI-adjusted WC | 40 | 0.097 (-0.135, 0.329) | 0.015 (-0.313, 0.344) | 0.802 (-0.382, 1.987) | 0.234 |
| BMI-adjusted WHR | 40 | 0.339 (0.114, 0.564) | 0.230 (-0.076, 0.536) | -0.332 (-1.489, 0.824) | 0.246 |
| BF percentage | 42 | 0.532 (0.162, 0.903) | 0.131 (-0.314, 0.577) | 0.129 (-1.715, 1.973) | 0.661 |
|  |  |  |  |  |  |
| **Female-specific** |  |  |  |  |  |
| BMI | 40 | 0.545 (0.232, 0.857) | 0.500 (0.179, 0.821) | 0.154 (-1.434, 1.741) | 0.622 |
| HC | 40 | 0.376 (0.016, 0.736) | 0.459 (0.131, 0.788) | 2.028 (0.202, 3.854) | 0.071 |
| WC | 39 | 0.461 (0.077, 0.845) | 0.464 (0.120, 0.808) | 1.413 (-0.564, 3.390) | 0.336 |
| WHR | 40 | 0.313 (-0.002, 0.627) | 0.427 (0.108, 0.747) | -0.021 (-1.679, 1.637) | 0.688 |
| BMI-adjusted HC | 40 | -0.005 (-0.261, 0.250) | -0.086 (-0.374, 0.201) | 1.300 (0.046, 2.554) | 0.038 |
| BMI-adjusted WC | 41 | 0.145 (-0.087, 0.377) | 0.278 (-0.003, 0.558) | 0.721 (-0.469, 1.910) | 0.334 |
| BMI-adjusted WHR | 41 | 0.148 (-0.123, 0.418) | 0.156 (-0.139, 0.451) | -0.515 (-1.903, 0.872) | 0.340 |
| BF percentage | 42 | 0.348 (0.027, 0.669) | 0.414 (-0.010, 0.838) | 0.724 (-0.882, 2.330) | 0.639 |

^†^: the SNPs used here were the SNPs left after removing those unmatched with the GWA outcome data and those associated with the outcome at significance level of 5*10^-8^. Therefore, these SNPs may contain those identified as outliers according to MR-PRESSO outlier tests. Table 2 shows the results using the SNPs after further removing outliers.

BMI: body mass index. WC: waist circumference. HC: hip circumference. WHR: waist-to-hip ratio. BF: body fat. ALM: appendicular lean mass. WLM: whole body lean mass. SNP: single nucleotide polymorphism.

Supplementary table 3. Results of multivariable Mendelian randomization analyses of sex-combined and sex-specific association between urinary sodium secretion with body mass, shape and composition outcomes, using all relevant SNPs^†^

|  |  | Inverse-variance weighted method | | |  | MR-Egger method | | | |  |
| --- | --- | --- | --- | --- | --- | --- | --- | --- | --- | --- |
| Outcome | SNP | beta | 95% CI | p for beta |  | beta | 95% CI | p for beta | p for Egger intercept | |
| **Sex-combined** | |  |  |  |  |  |  |  |  | |
| BMI | 40 | 0.399 | (0.095, 0.703) | 0.010 |  | -0.693 | (-2.198, 0.812) | 0.367 | 0.147 | |
| HC | 42 | 0.234 | (-0.105, 0.574) | 0.176 |  | 1.129 | (-0.526, 2.785) | 0.181 | 0.279 | |
| WC | 40 | 0.427 | (0.128, 0.726) | 0.005 |  | 0.142 | (-1.323, 1.606) | 0.85 | 0.696 | |
| WHR | 42 | 0.365 | (0.098, 0.632) | 0.007 |  | -0.24 | (-1.539, 1.059) | 0.717 | 0.351 | |
| BMI-adjusted HC | 42 | -0.135 | (-0.381, 0.110) | 0.280 |  | 1.104 | (-0.029, 2.237) | 0.056 | 0.028 | |
| BMI-adjusted WC | 43 | 0.121 | (-0.045, 0.287) | 0.154 |  | 0.719 | (-0.078, 1.516) | 0.077 | 0.133 | |
| BMI-adjusted WHR | 43 | 0.201 | (0.002, 0.400) | 0.048 |  | -0.492 | (-1.440, 0.455) | 0.308 | 0.143 | |
| BF percentage | 42 | 0.405 | (0.111, 0.699) | 0.007 |  | 0.433 | (-1.024, 1.891) | 0.560 | 0.969 | |
| WLM | 42 | 0.918 | (-0.549, 2.386) | 0.220 |  | -1.994 | (-9.378, 5.390) | 0.597 | 0.430 | |
| ALM | 42 | -0.069 | (-1.005, 0.867) | 0.885 |  | -2.142 | (-6.784, 2.499) | 0.366 | 0.371 | |
|  |  |  |  |  |  |  |  |  |  | |
| **Male-specific** | |  |  |  |  |  |  |  |  | |
| BMI | 41 | 0.396 | (0.037, 0.755) | 0.031 |  | -0.405 | (-2.164, 1.353) | 0.651 | 0.362 | |
| HC | 39 | 0.097 | (-0.304, 0.498) | 0.634 |  | 0.423 | (-1.701, 2.547) | 0.696 | 0.760 | |
| WC | 39 | 0.331 | (-0.095, 0.757) | 0.128 |  | 0.308 | (-1.957, 2.572) | 0.790 | 0.984 | |
| WHR | 39 | 0.511 | (0.182, 0.840) | 0.002 |  | -0.364 | (-2.086, 1.358) | 0.679 | 0.311 | |
| BMI-adjusted HC | 40 | -0.368 | (-0.719, -0.016) | 0.041 |  | 1.52 | (-0.188, 3.228) | 0.081 | 0.027 | |
| BMI-adjusted WC | 40 | 0.079 | (-0.156, 0.314) | 0.508 |  | 0.784 | (-0.402, 1.969) | 0.195 | 0.235 | |
| BMI-adjusted WHR | 40 | 0.321 | (0.094, 0.548) | 0.006 |  | -0.346 | (-1.503, 0.811) | 0.557 | 0.249 | |
| BF percentage | 42 | 0.538 | (0.159, 0.917) | 0.005 |  | 0.132 | (-1.734, 1.999) | 0.890 | 0.663 | |
|  |  |  |  |  |  |  |  |  |  | |
| **Female-specific** |  |  |  |  |  |  |  |  |  | |
| BMI | 40 | 0.53 | (0.211, 0.849) | 0.001 |  | 0.134 | (-1.468, 1.736) | 0.87 | 0.621 | |
| HC | 40 | 0.354 | (-0.013, 0.721) | 0.059 |  | 1.984 | (0.139, 3.829) | 0.035 | 0.078 | |
| WC | 39 | 0.423 | (0.037, 0.808) | 0.032 |  | 1.333 | (-0.636, 3.301) | 0.185 | 0.355 | |
| WHR | 40 | 0.269 | (-0.041, 0.579) | 0.089 |  | -0.11 | (-1.727, 1.508) | 0.894 | 0.640 | |
| BMI-adjusted HC | 40 | -0.017 | (-0.278, 0.244) | 0.900 |  | 1.278 | (0.009, 2.548) | 0.048 | 0.041 | |
| BMI-adjusted WC | 41 | 0.089 | (-0.127, 0.305) | 0.419 |  | 0.597 | (-0.498, 1.692) | 0.285 | 0.354 | |
| BMI-adjusted WHR | 41 | 0.097 | (-0.164, 0.359) | 0.466 |  | -0.623 | (-1.944, 0.698) | 0.356 | 0.276 | |
| BF percentage | 42 | 0.334 | (0.007, 0.662) | 0.045 |  | 0.713 | (-0.908, 2.334) | 0.388 | 0.640 | |

^†^: the SNPs used here were the SNPs left after removing those unmatched with the GWA outcome data and those associated with the outcome at significance level of 5*10^-8^. Therefore, these SNPs may contain those identified as outliers according to MR-PRESSO outlier tests. Table 3 shows the results using the SNPs after further removing outliers.

BMI: body mass index. WC: waist circumference. HC: hip circumference. WHR: waist-to-hip ratio. BF: body fat. ALM: appendicular lean mass. WLM: whole body lean mass. SNP: single nucleotide polymorphism.

Supplementary table 4. Results of univariable Mendelian randomization analyses of sex-combined and sex-specific association between urinary sodium secretion with body mass, shape and composition outcomes, using SNPs after removing MR-PRESSO outliers and rs1260236^†^

|  |  | Inverse-variance weighted method | | |  | Weighted median method | | |  | MR-Egger method | | | |
| --- | --- | --- | --- | --- | --- | --- | --- | --- | --- | --- | --- | --- | --- |
| Outcome | SNP | beta | 95% CI | p for beta |  | beta | 95% CI | p for beta |  | beta | 95%CI | p for beta | p for Egger intercept |
| **Sex-combined** |  |  |  |  |  |  |  |  |  |  |  |  |  |
| BMI | 34 | 0.392 | (0.149, 0.634) | 0.002 |  | 0.542 | (0.287, 0.797) | <0.001 |  | -0.193 | (-1.387, 1.001) | 0.751 | 0.327 |
| HC | 35 | 0.247 | (-0.014, 0.509) | 0.064 |  | 0.215 | (-0.080, 0.510) | 0.153 |  | -0.286 | (-1.703, 1.131) | 0.693 | 0.453 |
| WC | 37 | 0.508 | (0.228, 0.789) | <0.001 |  | 0.526 | (0.231, 0.822) | <0.001 |  | -0.041 | (-1.452, 1.370) | 0.954 | 0.436 |
| WHR | 37 | 0.467 | (0.227, 0.707) | <0.001 |  | 0.538 | (0.261, 0.815) | <0.001 |  | -0.006 | (-1.266, 1.254) | 0.993 | 0.453 |
| BMI-adjusted HC | 38 | -0.078 | (-0.245, 0.089) | 0.359 |  | 0.025 | (-0.210, 0.259) | 0.836 |  | 0.546 | (-0.270, 1.363) | 0.190 | 0.126 |
| BMI-adjusted WC | 42 | 0.138 | (-0.046, 0.323) | 0.142 |  | 0.137 | (-0.093, 0.367) | 0.242 |  | 0.630 | (-0.266, 1.525) | 0.168 | 0.272 |
| BMI-adjusted WHR | 39 | 0.253 | (0.061, 0.444) | 0.010 |  | 0.250 | (0.004, 0.496) | 0.046 |  | 0.152 | (-0.891, 1.195) | 0.775 | 0.848 |
| BF percentage | 40 | 0.531 | (0.261, 0.801) | <0.001 |  | 0.459 | (0.114, 0.803) | 0.009 |  | 0.299 | (-1.065, 1.664) | 0.668 | 0.734 |
| WLM | 40 | 0.639 | (-0.579, 1.857) | 0.304 |  | 0.831 | (-0.832, 2.495) | 0.327 |  | -3.710 | (-10.054, 2.634) | 0.252 | 0.171 |
| ALM | 40 | -0.233 | (-1.008, 0.542) | 0.556 |  | 0.034 | (-1.017, 1.086) | 0.949 |  | -3.363 | (-7.259, 0.533) | 0.091 | 0.108 |
|  |  |  |  |  |  |  |  |  |  |  |  |  |  |
| **Male-specific** |  |  |  |  |  |  |  |  |  |  |  |  |  |
| BMI | 38 | 0.417 | (0.113, 0.720) | 0.007 |  | 0.610 | (0.289, 0.931) | <0.001 |  | 0.378 | (-1.117, 1.874) | 0.620 | 0.959 |
| HC | 37 | 0.197 | (-0.155, 0.549) | 0.272 |  | 0.196 | (-0.193, 0.584) | 0.323 |  | 1.451 | (-0.394, 3.297) | 0.123 | 0.175 |
| WC | 35 | 0.476 | (0.157, 0.796) | 0.003 |  | 0.584 | (0.175, 0.993) | 0.005 |  | 0.646 | (-1.078, 2.370) | 0.463 | 0.844 |
| WHR | 37 | 0.698 | (0.408, 0.988) | <0.001 |  | 0.571 | (0.193, 0.948) | 0.003 |  | -0.589 | (-2.116, 0.938) | 0.450 | 0.093 |
| BMI-adjusted HC | 37 | -0.342 | (-0.654, -0.030) | 0.032 |  | -0.144 44 | (-0.517, 0.228) | 0.448 |  | 1.156 | (-0.450, 2.762) | 0.158 | 0.063 |
| BMI-adjusted WC | 39 | 0.149 | (-0.088, 0.385) | 0.217 |  | 0.184 | (-0.155, 0.524) | 0.287 |  | 1.150 | (-0.043, 2.342) | 0.059 | 0.094 |
| BMI-adjusted WHR | 39 | 0.340 | (0.108, 0.573) | 0.004 |  | 0.191 | (-0.152, 0.535) | 0.274 |  | -0.374 | (-1.578, 0.831) | 0.543 | 0.237 |
| BF percentage | 40 | 0.686 | (0.334, 1.038) | <0.001 |  | 0.307 | (-0.142, 0.755) | 0.180 |  | -0.107 | (-1.859, 1.646) | 0.905 | 0.365 |
|  |  |  |  |  |  |  |  |  |  |  |  |  |  |
| **Female-specific** |  |  |  |  |  |  |  |  |  |  |  |  |  |
| BMI | 36 | 0.571 | (0.295, 0.848) | <0.001 |  | 0.552 | (0.243, 0.860) | <0.001 |  | 0.011 | (-1.421, 1.443) | 0.988 | 0.434 |
| HC | 36 | 0.527 | (0.227, 0.826) | 0.001 |  | 0.58 | (0.252, 0.909) | 0.001 |  | 1.239 | (-0.401, 2.880) | 0.139 | 0.386 |
| WC | 32 | 0.517 | (0.214, 0.820) | 0.001 |  | 0.569 | (0.220, 0.917) | 0.001 |  | 0.144 | (-1.472, 1.760) | 0.861 | 0.645 |
| WHR | 36 | 0.367 | (0.074, 0.660) | 0.014 |  | 0.429 | (0.094, 0.764) | 0.012 |  | 0.571 | (-1.100, 2.243) | 0.503 | 0.808 |
| BMI-adjusted HC | 37 | -0.011 | (-0.226, 0.205) | 0.923 |  | -0.062 | (-0.365, 0.242) | 0.691 |  | 0.765 | (-0.360, 1.891) | 0.183 | 0.169 |
| BMI-adjusted WC | 39 | 0.140 | (-0.078, 0.357) | 0.208 |  | 0.235 | (-0.061, 0.532) | 0.119 |  | 0.274 | (-0.854, 1.402) | 0.634 | 0.812 |
| BMI-adjusted WHR | 37 | 0.186 | (-0.033, 0.404) | 0.096 |  | 0.160 | (-0.124, 0.445) | 0.269 |  | 0.654 | (-0.608, 1.915) | 0.310 | 0.460 |
| BF percentage | 41 | 0.383 | (0.051, 0.715) | 0.024 |  | 0.466 | (0.026, 0.905) | 0.038 |  | 0.939 | (-0.725, 2.604) | 0.269 | 0.504 |

^†^: the SNPs used here were the SNPs left after removing those unmatched with the GWA outcome data and those associated with the outcome at significance level of 5*10^-8^, and those identified as outliers by MR-PRESSO outlier test and rs1260326, which is associated with eGFR.

BMI: body mass index. WC: waist circumference. HC: hip circumference. WHR: waist-to-hip ratio. BF: body fat. ALM: appendicular lean mass. WLM: whole body lean mass. SNP: single nucleotide polymorphism.

Supplementary table 5. Results of univariable Mendelian randomization analyses of sex-combined and sex-specific association between urinary sodium secretion with body mass, shape and composition outcomes, using SNPs after removing MR-PRESSO outliers, rs1260236 and rs4803378^†^

|  |  | Inverse-variance weighted method | | |  | Weighted median method | | |  | MR-Egger method | | | |
| --- | --- | --- | --- | --- | --- | --- | --- | --- | --- | --- | --- | --- | --- |
| Outcome | SNP | beta | 95% CI | p for beta |  | beta | 95% CI | p for beta |  | beta | 95%CI | p for beta | p for Egger intercept |
| **Sex-combined** |  |  |  |  |  |  |  |  |  |  |  |  |  |
| BMI | 33 | 0.396 | (0.149, 0.644) | 0.002 |  | 0.576 | (0.309, 0.842) | <0.001 |  | -0.181 | (-1.467, 1.105) | 0.783 | 0.370 |
| HC | 34 | 0.248 | (-0.018, 0.515) | 0.068 |  | 0.237 | (-0.055, 0.528) | 0.112 |  | -0.344 | (-1.898, 1.209) | 0.664 | 0.448 |
| WC | 36 | 0.518 | (0.233, 0.802) | <0.001 |  | 0.549 | (0.251, 0.847) | <0.001 |  | 0.066 | (-1.456, 1.587) | 0.933 | 0.553 |
| WHR | 36 | 0.490 | (0.257, 0.724) | <0.001 |  | 0.546 | (0.283, 0.809) | <0.001 |  | 0.398 | (-0.924, 1.721) | 0.555 | 0.889 |
| BMI-adjusted HC | 37 | -0.086 | (-0.254, 0.082) | 0.318 |  | -0.004 | (-0.241, 0.232) | 0.971 |  | 0.479 | (-0.381, 1.339) | 0.275 | 0.189 |
| BMI-adjusted WC | 41 | 0.149 | (-0.036, 0.334) | 0.114 |  | 0.151 | (-0.082, 0.384) | 0.203 |  | 0.855 | (-0.067, 1.776) | 0.069 | 0.126 |
| BMI-adjusted WHR | 39 | 0.253 | (0.061, 0.444) | 0.010 |  | 0.250 | (0.004, 0.496) | 0.046 |  | 0.152 | (-0.891, 1.195) | 0.775 | 0.848 |
| BF percentage | 39 | 0.529 | (0.254, 0.804) | <0.001 |  | 0.440 | (0.097, 0.783) | 0.012 |  | 0.235 | (-1.239, 1.709) | 0.754 | 0.691 |
| WLM | 39 | 0.729 | (-0.494, 1.951) | 0.243 |  | 0.833 | (-0.859, 2.525) | 0.335 |  | -2.397 | (-9.081, 4.287) | 0.482 | 0.351 |
| ALM | 39 | -0.177 | (-0.944, 0.591) | 0.652 |  | 0.053 | (-0.961, 1.067) | 0.918 |  | -2.650 | (-6.780, 1.479) | 0.208 | 0.232 |
|  |  |  |  |  |  |  |  |  |  |  |  |  |  |
| **Male-specific** |  |  |  |  |  |  |  |  |  |  |  |  |  |
| BMI | 37 | 0.414 | (0.105, 0.723) | 0.009 |  | 0.643 | (0.326, 0.960) | <0.001 |  | 0.333 | (-1.284, 1.949) | 0.687 | 0.920 |
| HC | 37 | 0.197 | (-0.155, 0.549) | 0.272 |  | 0.196 | (-0.193, 0.584) | 0.323 |  | 1.451 | (-0.394, 3.297) | 0.123 | 0.175 |
| WC | 35 | 0.476 | (0.157, 0.796) | 0.003 |  | 0.584 | (0.175, 0.993) | 0.005 |  | 0.646 | (-1.078, 2.370) | 0.463 | 0.844 |
| WHR | 37 | 0.698 | (0.408, 0.988) | <0.001 |  | 0.571 | (0.193, 0.948) | 0.003 |  | -0.589 | (-2.116, 0.938) | 0.450 | 0.093 |
| BMI-adjusted HC | 37 | -0.342 | (-0.654, -0.030) | 0.032 |  | -0.144 | (-0.517, 0.228) | 0.448 |  | 1.156 | (-0.450, 2.762) | 0.158 | 0.063 |
| BMI-adjusted WC | 39 | 0.149 | (-0.088, 0.385) | 0.217 |  | 0.184 | (-0.155, 0.524) | 0.287 |  | 1.150 | (-0.043, 2.342) | 0.059 | 0.094 |
| BMI-adjusted WHR | 39 | 0.340 | (0.108, 0.573) | 0.004 |  | 0.191 | (-0.152, 0.535) | 0.274 |  | -0.374 | (-1.578, 0.831) | 0.543 | 0.237 |
| BF percentage | 39 | 0.674 | (0.318, 1.031) | <0.001 |  | 0.244 | (-0.212, 0.701) | 0.294 |  | -0.424 | (-2.289, 1.441) | 0.656 | 0.240 |
|  |  |  |  |  |  |  |  |  |  |  |  |  |  |
| **Female-specific** |  |  |  |  |  |  |  |  |  |  |  |  |  |
| BMI | 35 | 0.584 | (0.305, 0.862) | <0.001 |  | 0.564 | (0.254, 0.874) | <0.001 |  | 0.187 | (-1.341, 1.714) | 0.811 | 0.604 |
| HC | 36 | 0.527 | (0.227, 0.826) | 0.001 |  | 0.580 | (0.252, 0.909) | 0.001 |  | 1.239 | (-0.401, 2.880) | 0.139 | 0.386 |
| WC | 32 | 0.517 | (0.214, 0.820) | 0.001 |  | 0.569 | (0.220, 0.917) | 0.001 |  | 0.144 | (-1.472, 1.760) | 0.861 | 0.645 |
| WHR | 36 | 0.367 | (0.074, 0.660) | 0.014 |  | 0.429 | (0.094, 0.764) | 0.012 |  | 0.571 | (-1.100, 2.243) | 0.503 | 0.808 |
| BMI-adjusted HC | 37 | -0.011 | (-0.226, 0.205) | 0.923 |  | -0.062 | (-0.365, 0.242) | 0.691 |  | 0.765 | (-0.360, 1.891) | 0.183 | 0.169 |
| BMI-adjusted WC | 39 | 0.140 | (-0.078, 0.357) | 0.208 |  | 0.235 | (-0.061, 0.532) | 0.119 |  | 0.274 | (-0.854, 1.402) | 0.634 | 0.812 |
| BMI-adjusted WHR | 37 | 0.186 | (-0.033, 0.404) | 0.096 |  | 0.160 | (-0.124, 0.445) | 0.269 |  | 0.654 | (-0.608, 1.915) | 0.310 | 0.460 |
| BF percentage | 40 | 0.390 | (0.052, 0.728) | 0.024 |  | 0.487 | (0.053, 0.921) | 0.028 |  | 1.146 | (-0.644, 2.936) | 0.210 | 0.399 |
